# Microstructure, and Mechanical and Wear Properties of Grp/AZ91 Magnesium Matrix Composites

**DOI:** 10.3390/ma12071190

**Published:** 2019-04-11

**Authors:** Chang-rui Wang, Kun-kun Deng, Yan Bai

**Affiliations:** 1 College of Mechanical and Electrical Engineering, Nanjing University of Aeronautics and Astronautics, Nanjing 210016, China; wchrui2016@163.com; 2 School of Materials Science and Engineering, Taiyuan University of Technology, Taiyuan 030024, China; 35645105@163.com; 3 The 14th Research Institute of China Electronics Technology Group Corporation, Nanjing 210039, China

**Keywords:** magnesium matrix composites, thermal deformation, microstructure, mechanical property, wear mechanism

## Abstract

Based on semi-solid mixing technology, two kinds of as-cast Grp (Graphite particles)/AZ91 composites with different Grp volume fractions (5 vol %, 10 vol %) were prepared; these are called 5 vol % Grp/AZ91 composites and 10 vol % Grp/AZ91 composites, respectively. In order to eliminate casting defects, refine grains, and improve mechanical properties, thermal deformation analysis of these composites was conducted. The effect of the addition of Grp and thermal deformation on the microstructure, mechanical properties, and wear resistance of AZ91 composite was explored. The results showed that after 5 vol % Grp was added into the as-cast AZ91 alloy, Mg_17_Al_12_ phases were no longer precipitated reticularly along the grain boundary, and Al_4_C_3_ phases were formed inside the composite. With the increase in the volume fraction of Grp, the grains of the AZ91 composites were steadily refined. With the increase of forging pass, the grain size of 5% Grp/AZ91 composites decreased first, and then increased. Additionally, the Grp size decreased gradually. There was little change in the yield strength, and the tensile strength and elongation were improved to a certain extent. After forging and extrusion of 5% Grp/AZ91 composites once, the grain size and Grp size were further reduced, and the yield strength, tensile strength, and elongation were increased by 23%, 30%, and 65%, respectively, compared with the composite after forging. With the increase of the number of forging passes before extrusion, the grain size decreased little by little, while the Grp size remained unchanged. The average yield strength, tensile strength, and elongation of the composites after forging and extrusion six times were increased by 3%, 3%, and 23%, respectively, compared with the composite after forging and extrusion once. The wear rate and friction coefficient of the 5% Grp/AZ91 composites decreased after forging once, and the wear mechanism was mainly due to ploughing wear. By comparison, the wear rate and friction coefficient of the 5% Grp/AZ91 composites increased in the extrusion state, and the main wear mechanism was from wedge formation and micro-cutting wear.

## 1. Introduction

With the development of miniaturization, and high integration of aerospace, automotive, and electronic equipment, higher requirements for lightweight structural materials have been put forward. As the lightest structural materials in engineering applications, magnesium (Mg) and its alloys have the characteristics of low density, high specific strength, specific stiffness, and good electromagnetic shielding, and they also have the advantages of good recyclability, easy turning, and good casting performance; thus, they are known as “green structural materials in the 21st century” [1,2,3,4]. AZ91 magnesium alloy is one of the most widely used casting magnesium alloys in industry. A and Z refer to aluminium(Al) and zinc(Zn), and the 91 refers to that the nominal content of AlZn is 9 wt % and 1 wt % respectively. However, the application of Mg alloys in engineering materials is limited, due to their low modulus, low strength, low hardness, poor wear resistance, high thermal expansion coefficient, and poor high-temperature resistance. Mg matrix composites that are reinforced by adding particles and whiskers feature a good specific modulus, specific strength, wear resistance, adjustable linear expansion coefficient, and so on. Therefore, they have great potential applications in aerospace, military electronics, automotive, orbital, and other fields [5,6,7,8,9]. Commonly used reinforcing particles and whiskers include: SiCp [10], TiCp [11], B_4_Cp [12], SiCw [13], Ce [14], etc. There is a small interlaminar Van der Waals force in Grp, and it is easy for them to form a large coverage lubrication layer on the friction surface. By adding graphite into the composite, the lubrication conditions of friction pairs can be improved [15]. In addition, it was proven that hot extrusion could improve the distribution of particles in the matrix and the comprehensive mechanical properties of the material. Therefore, materials with both good mechanical properties and wear resistance can be obtained by adding reinforcement particles into magnesium composites by means of hot extrusion [16].

The mechanical properties and wear resistances of particle-reinforced metal matrix composites have been studied. Meng et al. [17] studied the effect of the addition of Bi on the microstructure and mechanical properties of magnesium matrix composites. The mechanical properties were significantly improved. Lim et al. [18] studied the effect of load and sliding velocity on the wear resistance and wear mechanism of SiCp-reinforced magnesium matrix composites under dry friction. The composites reinforced by SiC particulate exhibited slightly superior wear resistance under a lower load. However, the effects of the reinforcements on wear resistance were not as conclusive under a higher load. Huang et al. [19] studied the effects of the volume fraction and size of micron SiCp on the wear rate, hardness, and friction coefficients of magnesium matrix composites. Liu et al. [20] indicated that the hardness increased with the increase in Y contents, due to the uniform distribution of the Al_2_Y and Mg_17_Al_12_ phases. Luo et al. [21] indicated that with the increase of graphite content, the friction coefficient and wear rate of the Al–Zn alloy decreased. Akhlaghi et al. [22] demonstrated that the wear resistances of Al 2024-Grp composites under dry friction first increased, and then decreased with the increase of Grp content. However, the mechanical properties and wear resistances of extruded Grp-reinforced magnesium matrix composites have seldom been studied. 

In this paper, a semi-solid stirring technique was employed to design and fabricate as-cast Grp/AZ91 composites. Then, multi-directional forging (MDF) and hot extrusion deformation (ED) were carried out on as-cast Grp/AZ91 composites. The effects of Grp on the microstructure, mechanical properties, and wear resistances of as-cast, forged, and extruded magnesium matrix composites were investigated. The microstructure evolution, mechanical properties, and strengthening mechanisms after hot deformation were studied.

## 2. Experimental Methods

### 2.1. Sample Preparation

The AZ91 alloy features a wide, semi-solid temperature range, and good casting properties and mechanical properties, and it is an ideal matrix for preparing magnesium matrix composites by semi-solid stirring. The matrix alloy used in the experiment was AZ91 alloy without particle reinforcement phases (hereafter called AZ91 alloy). The main chemical compositions of the AZ91 alloys were Mg, Al, and Zn. In addition, it contained a small amount of Si, Mn, Cu, Ni, and Fe. The mass fractions of various elements in the AZ91 alloy (wt %) are shown in Table 1. 

**Figure 1:**
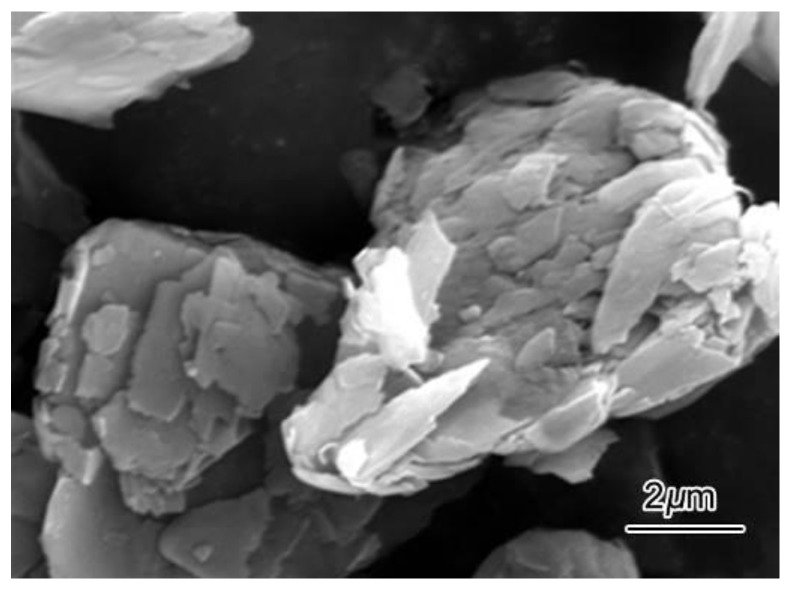
SEM image of particle morphology for Grp.

Graphite was used in the experiment as the particle reinforcement phase. The average size of the high-purity fine graphite particles used in the test was 5 μm, which was provided by Qingdao Furunda Graphite Co., Ltd. The scanning electron microscopy (SEM) morphology of the graphite particles is shown in Figure 1. Many of studies found that Ca addition affected the solidification process of AZ91 alloy, which could refine grain size and improve mechanical properties [23,24,25]. More importantly, a small amount of Ca addition could prevent oxidation in the preparation of as-cast Grp/AZ91 composites reinforced by Grp (hereafter called AZ91 composites). Therefore, 0.5 wt % Ca (AZ91 alloy: Ca = 99.5 wt %:0.5 wt %) was added during the preparation of AZ91 composites.

The preparation process of the AZ91 composites was as follows. First, the Grp were cleaned, dried, and sifted to remove impurities, such as SiO_2_ on the surface of the particles, and to ensure that there were no large agglomerations between the particles. The specific operation was as follows: Grp was added to distilled water, stirred for 5 min, and then set aside. After Grp sank completely, the upper liquid was poured out, so that it could be reciprocated three times. Subsequently, the washed Grp was placed in the drying box, dried at 80 °C for 720 min, then heated to 120 °C, and drying continued for 120 min. Second, the surface of the AZ91 alloy were ground with a sand grinder and coarse sandpaper to make the composite surface clean and free of impurities. Finally, tools such as a crucible, mold, and stirring slurry were cleaned up to avoid contamination of the composite fluid.

The cleaned AZ91 alloy was put into the crucible, with the covering agent being evenly sprayed on the surface. The brand of covering agent used in this experiment was RJ-6. The specific composition is shown in Table 2. The temperature of the resistance furnace was set to 780 °C, and a mixture of CO_2_ and SF_6_ (CO_2_:SF_6_ = 40:1 volume ratio) was introduced. When the AZ91 alloy was fully melted, the resistance furnace temperature was lowered to 635 °C (the semi-solid temperature of the AZ91 alloy). The AZ91 alloy was then stirred until a clear and stable eddy current appeared (forming a completed eddy current shape) in the semi-solid slurry.

Grp with an average size of 5 μm was added to the stirring melt and preheated to 450 °C in advance. After adding Grp, the AZ91 composite was stirred at a high speed (600 rpm) for 20 min. The resistance furnace was then heated to 780 °C, with the stirring speed being gradually reduced with the increase of temperature. When the temperature reached 780 °C, the resistance furnace stopped stirring and it was kept warm for 5 min. Finally, the melt was poured into the mold and solidified at 400 °C and 450 MPa (mould pressure). 

To improve their properties, the as-cast Grp/AZ91 composites were subjected to forging and hot extrusion processes several times. The forging process was as follows: first, the as-cast composites were processed by wire cutting into a cubic specimen (30 mm × 30 mm × 60 mm), which were then cleaned with sandpaper. Next, both the specimen and the forging die were preheated to 400 °C. After the preheating was completed, parameters were set, and the forging was commenced. The loading speed was 5 kN/s, and the pressure was maintained at 450 MPa for 180 s, so that the height of the specimen was reduced by about 50%. The next stage was to take out the specimen, rotate it to 90°, and subject it to a second forging process along its long-axis direction, to allow the height of the specimen to be reduced by about 50%. These steps were repeated to prepare composites that were forged once, three times, and six times, successively. The hot extrusion process was as follows: (1) The composite that was forged once was homogenized (415 °C + 24 h), and then cleaned; (2) the specimen and die were preheated to 300 °C, and the temperature was kept for 20 min; (3) the preheated specimen was placed in an extrusion die with an extrusion ratio of 16:1 for extrusion at a speed of 0.05 mm/s, and then extrusion was commenced. After the extrusion was completed, a composite bar with a diameter of 10 mm was obtained; (4) the above steps were repeated to complete the hot extrusion of other two forged composites.

### 2.2. Test Methods

The microstructure, tensile fracture, and friction and wear surfaces of the composites were observed by using a 4XC optical microscope (OM) (Shang Guang, Shanghai, China) and a MIRA 3XMU70 SEM (TESCAN, Brno, Czech Republic), and its accompanying energy disperse spectroscopy (EDS). An Instron 5569 (Instron, Boston, USA) testing machine was used to conduct a tensile test on the composites at a constant speed of 0.5 mm/min. The tensile direction was parallel to the extrusion direction, the gauge length of the specimen was 15 mm, and the cross section was 6 mm × 2 mm. Three specimens were tested for each composite, and the average of their tensile properties was taken. Friction and wear tests were carried out on a specimen whose size was 8 mm in diameter and 20 mm in length, by using a ML-10 pin-disc friction (Cheng Xin, Zhangjiakou, China) and a wear testing machine at a load of 500 g, at room temperature. The specific test parameters were as follows: (1) Disc diameter: 240 mm; (2) disc speed: 300 rpm; (3) sliding speed: 1 mm/s; (4) the friction media were: different types of abrasive cloths (included 400#, 800#, 1000#, and 1500#). The specimen was cleaned with alcohol before and after the test, and then weighed with a JA2003 type electronic balance (with the precision of 10^−4^ g). The wear rate was calculated by measuring the mass loss. The friction coefficient test was performed using the MFT-R400 reciprocating friction and wear testing machine (Heng Xu, Jinan, China) at a normal load of 3 N, a reciprocating frequency of 2 Hz, a stroke of 5 mm, and at room temperature. The GCr15 steel ball with a diameter of 5 mm was used as the friction pair.

## 3. Results and Discussion

### 3.1. As-cast Microstructure of the AZ91 Alloy and the Grp/AZ91 Composites

**Figure 2:**
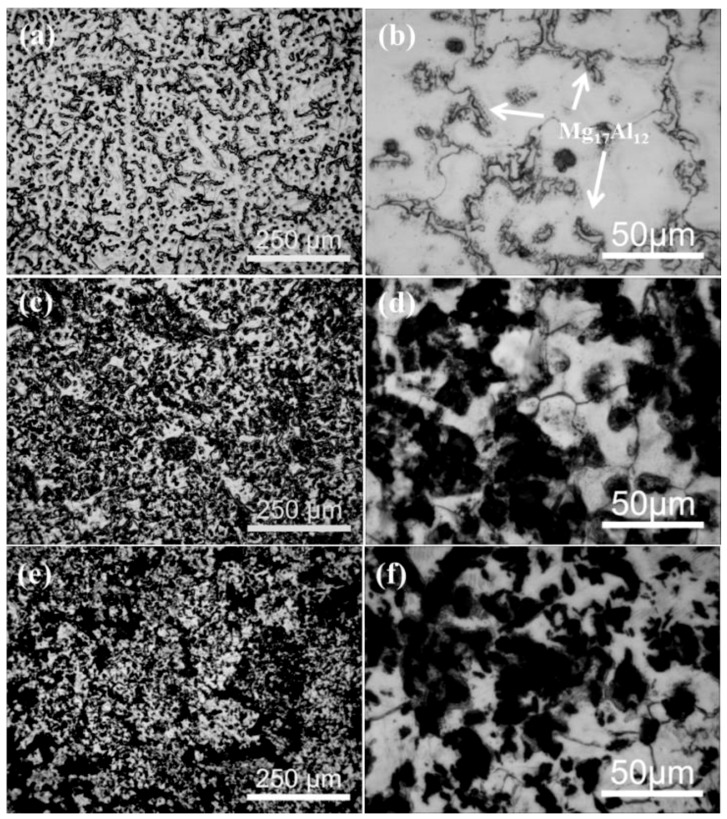
Low- (**a**,**c**,**e**) and high- (**b**,**d**,**f**) magnification OM images of the as-cast Grp/AZ91 composites: AZ91 alloy (**a**,**b**); 5% Grp/AZ91 composites (**c**,**d**); 10% Grp/AZ91 composites (**e**,**f**).

Figure 2 shows the optical microstructure of the as-cast AZ91 alloy and the Grp/AZ91 composite. As can be seen from Figure 2a,b, the grains of the as-cast AZ91 alloys were large in size. The second phase, i.e., the Mg_17_Al_12_ phase, was mainly precipitated reticularly along the grain boundary, and a small part of it was distributed in the matrix in granular form. This distribution pattern along the grain boundary impaired the mechanical properties of the matrix. Due to the high energy at the grain boundary, the Al element in the AZ91 alloy in the form of a solid solution tended to be concentrated here, to form the Mg_17_Al_12_ phase [26,27]. After the addition of 5 vol % of Grp, as shown in Figure 2c,d, the grain size of the Grp/AZ91 composites decreased, and the distribution of Grp was fairly uniform. The precipitates (Mg_17_Al_12_) at the grain boundaries almost entirely disappeared. The introduction of the 5 vol % Grp effectively improved the distribution of the second phase in the AZ91 composite. Figure 2e,f show the Grp/AZ91 composites with 10 vol % Grp added. The grain size of the 5% Grp/AZ91 composite further decreased, but to just a small degree. These suggested that the addition of Grp to 10 vol % did not make the distribution of the second phase change significantly, though slight particle agglomeration was observed. The OM images of the as-cast AZ91 alloy, 5% Grp/AZ91 composites, and 10% Grp/AZ91 composites, were statistically analyzed by Image-Pro Plus, and the average grain sizes of the three composites were 95.1 μm, 79.3 μm, and 72.4 μm, respectively. As the volume fraction of Grp increased, the grain size was gradually refined. This is because a higher graphite content produced a greater number of heterogeneous nuclear particles in the matrix composite, thereby increasing the nucleation rate and leading to grain refinement, which was conducive to the improvement of the mechanical properties of the composite.

**Figure 3:**
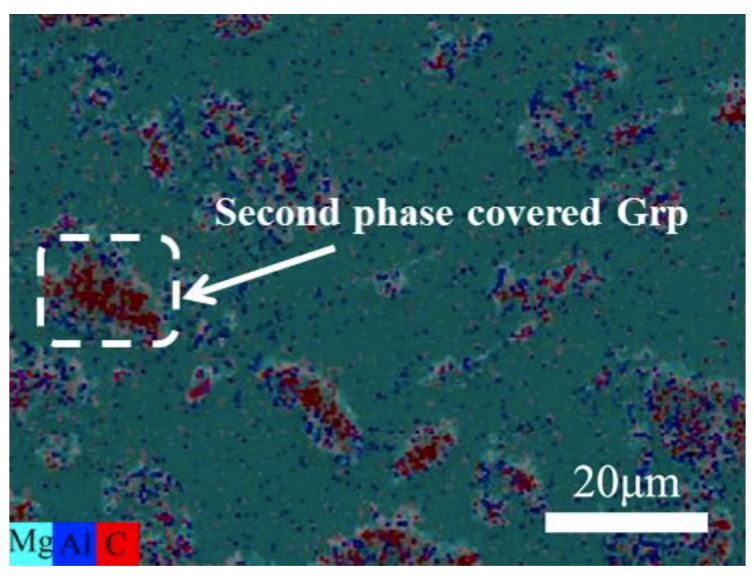
EDS of as-cast 5% Grp/AZ91 composites.

**Figure 4:**
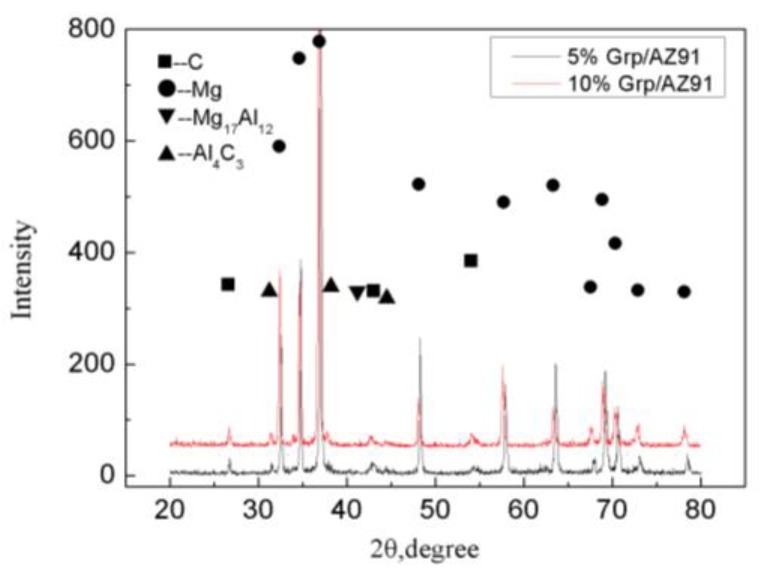
The X-ray diffraction of the as-cast Grp/AZ91 composites.

Figure 3 shows the surface scanning of the as-cast 5% Grp/AZ91 composites. It shows that most of the second phases containing Al accumulated at the interface between the graphite particles and the matrix. This indicated that the addition of Grp effectively inhibited the precipitation of the Mg_17_Al_12_ phase at the grain boundary. From the X-ray diffraction (XRD) analysis of the as-cast 5% Grp/AZ91 composites and the 10% Grp/AZ91 composites (Figure 4), the diffraction peak of the Mg_17_Al_12_ phase in the as-cast 5% Grp/AZ91composites was small in size, consistent with the analysis of the microstructure. The appearance of the diffraction peak of the Mg_17_Al_12_ phase in the 10% Grp/AZ91 composites revealed that the addition of Grp was harmful to the microstructure and properties of the composite. Moreover, besides the Mg_17_Al_12_ phase, the Al_4_C_3_ phases were found in the two composites.

The reason for the appearance of Al_4_C_3_ phase in the composite was the occurrence of the interface reaction. The study of Wang et al. [28] found that Al powder and C powder could react rapidly above 520 °C to form the Al_4_C_3_ phase. Lu et al. [29] found the formation of Al_4_C_3_ when conducting heat preservation on the graphite particle reinforced the aluminum matrix composite at 630 °C. In this test, the casting temperature may exceed 700 °C, and the AZ91 composite contained 9.3 wt % of Al, and the AI could react with Grp to form the Al_4_C_3_ phase in the process of casting. Based on this, the addition of 5% Grp was beneficial for the microstructure and properties of the AZ91 composite.

### 3.2. Effect of Multiple Forging on Microstructure of Grp/AZ91 Composites

**Figure 5:**
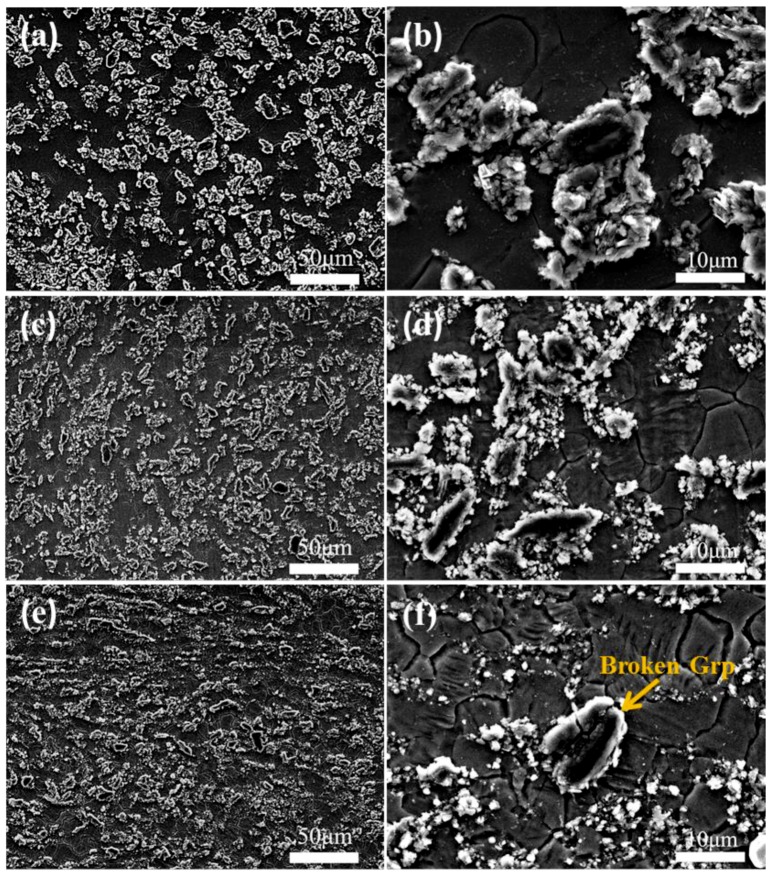
Low- (**a**,**c**,**e**) and high- (**b**,**d**,**f**) magnification SEM images of 5% Grp/AZ91 after MDF: 1FD (**a**,**b**); 3FD (**c**,**d**); 6FD (**e**,**f**).

On the basis of the casting composites, considering that the mechanical properties of the as-cast composites were not ideal, it was necessary to conduct thermal deformation to improve their properties. Thermal deformation can not only eliminate casting defects, but also refine the grains, making the distribution of the reinforcing phases in the composite more uniform. Figure 5 shows the SEM image of the 5% Grp/AZ91 composites after MDF under different forging deformation times (FD) (1FD, 3FD, 6FD represent one, three and six MDFs respectively). It can be seen from Figure 5a that the Grp distribution in the 5% Grp/AZ91 composites were more uniform after forging once, and the slight particle agglomeration phenomenon in the as-cast composite was eliminated, and the average size of Grp was reduced. Figure 5c,d are SEM images of 5% Grp/AZ91 after forging three times, and the Grp was more broken and refined than Grp of the composite after forging once. Figure 5e,f are SEM images of 5% Grp/AZ91 after forging six times. The size of Grp further decreased, and obvious cracks could be observed inside the Grp under high-magnification SEM. This was because after forging six times, high stress was accumulated on Grp, while Grp itself had relatively low strength, leading to the rupture of Grp.

The size of Grp was statistically analyzed by Image-Pro Plus software, as shown in Table 3. For the size values in Table 3, the number before ‘±’ is the average value, and the number after ‘±’ is the standard deviation. The standard deviation was larger, with the grain size being more uneven. The size of Grp decreased with the increase of the forging deformation frequency, which was consistent with the results of the microstructure observed in SEM. Wu et al. [30,31] studied the hot extrusion of a 50 μm Grp-reinforced magnesium matrix composite, and found that the aspect ratio of Grp increased monotonously with the increase of extrusion temperature. In this test, there was no obvious increase in the aspect ratio of Grp, but Grp breakage occurred. This is due to the following reasons: (1) The extrusion temperature was lower than the extrusion temperature in the test of Wu, which is not conducive to the relative slip of the graphite sheet; (2) Grp is small in size and prone to plastic flow with the matrix during extrusion; (3) the Al_4_C_3_ phase with higher hardness wrapped at the Grp interface did not match Grp deformation during extrusion, and as a result, the Grp broke into finer particles. Combined with the above analysis, we could see that the change of Grp size was caused by particle breakage during deformation. The average size of the particles was smaller than the deviation, which indicated that there were more small particles and fewer large ones.

The grain sizes of the 5% Grp/AZ91 composites were statistically analyzed by Image-Pro Plus software, and the results are shown in Table 4. It can be seen that after forging once, the grain size was obviously refined. Compared with the grain size of the as-cast composite, the degree of recrystallization was relatively complete, and there were no coarse grains that had not yet undergone recrystallization. Nie et al. [32] reported that after multi-directional forging at 400 °C, there were still many areas in the microstructure of the AZ91 composite without recrystallization, and this indicated that the graphite particles could promote the occurrence of recrystallization. The change of grain size was caused by dynamic recrystallization (DRX). During the forging process, the occurrence of DRX resulted in grain refinement and uneven grain size. With the increase of forging passes, the degree of DRX and the uniformity of grain size increased, and so the standard deviation decreased. The same was true for Grp size. With the increase of forging passes, the average particle size decreased and the standard deviation decreased. After forging three times, the grain size of the composite decreased slightly, but this increased after forging six times. This was because, on the one hand, with the increase of the number of forges, the initial microstructure before forging was more uniform, the grains and graphite particles were more refined, and there were more grain boundaries and interfaces, so that the recrystallization nucleation rate of the next forging increased. On the other hand, with the increase of forging frequency, the amount of strain accumulating in the composite increased, and the recrystallization driving force increased accordingly. The combined action of the recrystallization nucleation rate and driving force made the grain sizes of the 5% Grp/AZ91 composites first decrease, and then increase with the increase of forging rounds.

### 3.3. Effect of Multi-Step Deformation on the Microstructure of Grp/AZ91 Composites

**Figure 6:**
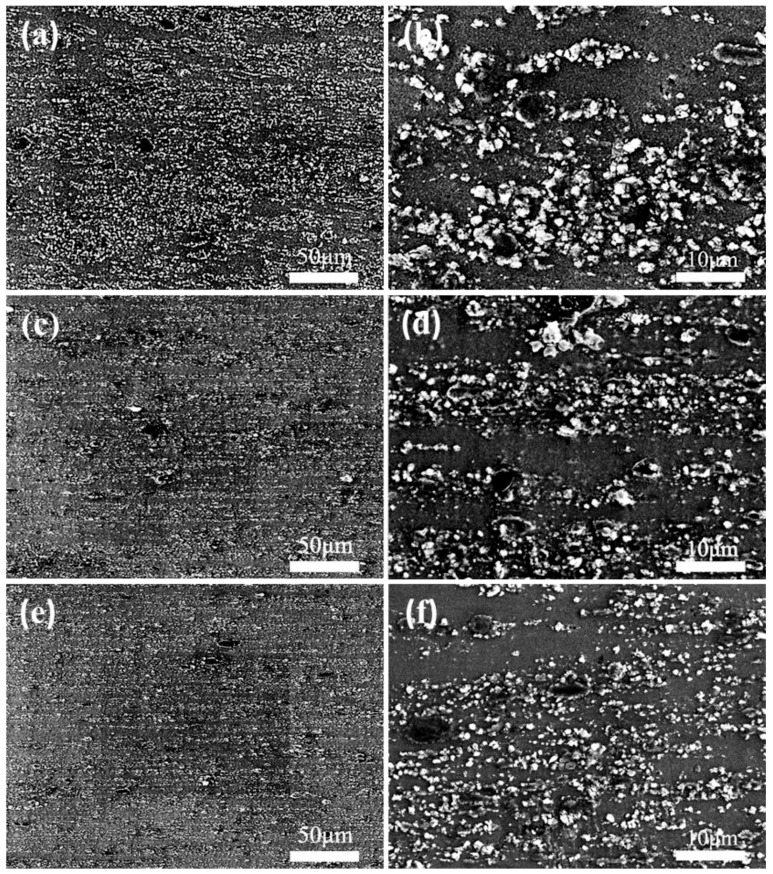
Low- (**a**,**c**,**e**) and high- (**b**,**d**,**f**) magnification SEM images of 5% Grp/AZ91 composites fabricated by multistep deformation: 1FD+ED (**a**,**b**); 3FD+ED (**c**,**d**); 6FD+ED (**e**,**f**).

Figure 6 shows the SEM image of the microstructure of the 5% Grp/AZ91 composites after multi-step deformation. 1FD+ED, 3FD+ED, 6FD+ED represent one MDF plus extrusion once, three MDFs plus extrusion once and six MDFs plus extrusion once, respectively.It can be seen from Figure 6a,b that compared with the composites after forging once, the Grp of the 5% Grp/AZ91 composites after forging plus extrusion once (1FD+ED) was distributed along the extrusion direction, and its size also significantly decreased. Figure 6c–f shows SEM images of the microstructures of the 5% Grp/AZ91 composites after forging plus extrusion three times and forging plus extrusion six times, respectively. It can be seen that compared with the 5% Grp/AZ91 composite after forging plus extrusion once, there was no significant change in the distribution and size of Grp of the 5% Grp/AZ91 composites after forging plus extrusion three times or forging plus extrusion six times.

The size of Grp in the composite after multi-step deformation was statistically analyzed by Image-Pro Plus software. The results are shown in Table 5. It can be found that with the increase of the forging times before extrusion, there was no obvious change in the size of Grp in the composite after extrusion. This was because, compared with extrusion deformation in multi-directional forging, extrusion deformation with an extrusion ratio of 16:1 was obviously more severe, having a stronger crushing effect on Grp. As a result, composites with different initial sizes of Grp shared nearly the same sizes of Grp after hot extrusion.

The grain sizes of the composites after multi-step deformation were statistically analyzed, and the results are shown in Table 6. It can be found that the grain sizes of the composites after multi-step deformation were obviously refined, compared with the grain sizes of the composites after multi-directional forging. This was because the recrystallization grains were refined due to large deformation during hot extrusion, the large driving force of recrystallization nucleation, and the large increase of the nucleation rate. In addition, with the increase of the forging frequency before extrusion, the grain size of the composite after extrusion decreased slightly. This was because the composite after forging was homogenized before extrusion, eliminating all possible influences of grain size and the Mg_17_Al_12_ phase. Before hot extrusion, there was only a difference in the size of Grp between composites with different forging times, and with the increase of forging times before extrusion, Grp was smaller in size and larger in relative quantity, which could obviously improve the recrystallization nucleation rate. Therefore, the composite after forging plus extrusion six times contained the finest grain.

### 3.4. The Effect of Multi-Directional Forging on the Mechanical Properties of Grp/AZ91 Composites

Table 7 shows the mechanical properties of the 5% Grp/AZ91 composite after multi-directional forging. It can be found that with the increase of forging times, there was no great change in the yield strength of the composite. The composite with the finest grain size after forging three times should have the highest yield strength. The reason for this phenomenon was that in the process of multi-directional forging, with the increase of forging passes, the axial direction of the applied load changed continuously, and the texture of the composite softened, so that the Schmidt factor of the base plane increased. As a result, the basal slip was easier to commence, and the yield strength decreased. It could also be found that with the increase of forging times, the tensile strength and elongation of the composite also increased. This was because on the one hand, some casting defects that might remain inside the composite were gradually eliminated; on the other hand, Grp was refined, the distribution of the particles was more uniform, and the particle segregation area was reduced. Additionally, the crack was not easily initiated or expanded. In addition, the microhardnesses of the 5% Grp/AZ91 composites increased first, and then decreased with the increase of forging frequency, and this was consistent with the change rule of the yield strengths of the composites.

### 3.5. Effect of Multi-Step Deformation on the Mechanical Properties of the Grp/AZ91 Composites

Table 8 shows the mechanical properties of the 5% Grp/AZ91 composites after multi-step deformation. It can be found that the mechanical properties of the composite after multi-step deformation were superior to the mechanical properties of the composite after multi-directional forging. Compared with the composites after forging once, the yield strength, tensile strength, and elongation of the composite after forging plus one round of extrusion were increased by 23%, 30%, and 65%, respectively. This was attributed to the more uniform microstructure and the fine recrystallization grain of the 5% Grp/AZ91 composites after multi-step deformation. By a longitudinal comparison of the mechanical properties of the three different composites after multi-step deformation in Table 7, it can be found that with the increase of forging frequency before extrusion, the yield strength, tensile strength, and elongation of the composites increased slightly, but the increase was not obvious. Compared with the composites after forging plus one round of extrusion, the yield strength, tensile strength, and elongation of the composite after forging plus extrusion six times were increased by 3%, 3%, and 23%, respectively. This may be attributed to the relatively fine grain size of the composite after forging plus extrusion six times.

**Figure 7:**
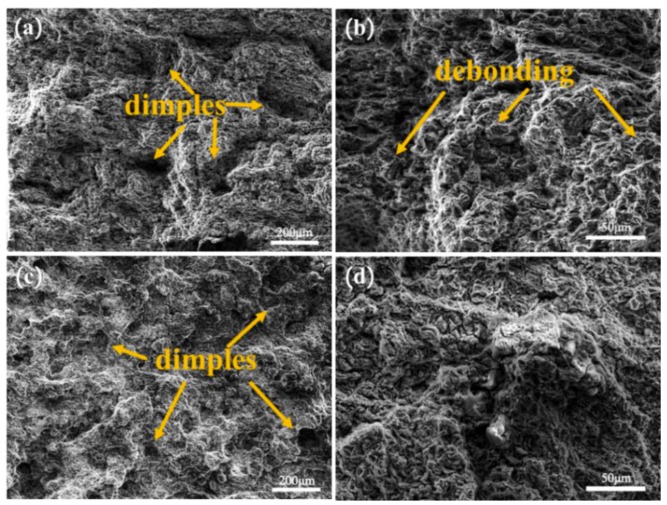
Low- (**a**,**c**) and high- (**b**,**d**) magnification SEM images of 5% Grp/AZ91 composites after hot deformation: 1FD (**a**,**b**); 1FD+ED (**c**,**d**).

Figure 7 shows the SEM images of the fracture of the 5% Grp/AZ91 composites after forging once, and the 5% Grp/AZ91 composites after forging plus extrusion once. From Figure 7a, it can be observed that the dimples on the matrix after forging once were large in size and small in quantity, which indicates that there was a small strain on the fracture surface, and that it had a typical brittle fracture morphology. In the high-magnification SEM image (Figure 7b), many Grp-sized pits and protrusions could be found, and this indicates that interface debonding occurred between the Grp and the matrix, and that the crack extended along the Grp interface, leading to fracture on the composite. From Figure 7c, it can be found that the dimples on the matrix after extrusion were small in size and large in quantity, indicating that much plastic deformation occurred before fracture. No obvious interface debonding was observed in the high-magnification SEM image (Figure 7d), indicating that the bonding strength of Grp against the matrix was increased after extrusion, and that the crack tended to be generated from the inside of the graphite particles, and that the cracking spread during subsequent stretching.

### 3.6. Wear resistance Analysis of Grp/AZ91 Composites

**Figure 8:**
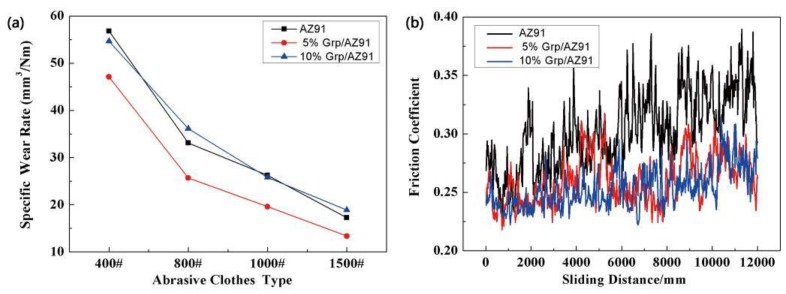
(**a**) Wear rate with different abrasive cloths, and (**b**) the friction coefficient versus time of the as-cast AZ91 alloy and Grp/AZ91 composites.

Figure 8a shows the change of wear rate of the as-cast AZ91 alloy and Grp/AZ91 composites, with the decrease of the abrasive cloth grit. With the decrease of the abrasive cloth grit, the wear rate of the AZ91 alloy and the Grp/AZ91 composites decreased. This was because, when the abrasive cloth grit was larger in size, the real contact area was smaller. As a result, plastic deformation more easily occurred on the soft matrix, and it was not conducive to the formation of the graphite lubrication layer. Compared with the as-cast AZ91 alloy, the wear rate of the Grp/AZ91 composite significantly decreased after adding 5 vol % of Grp. This was due to the following reasons: (1) After the addition of Grp, the hardness of the composite was improved, and the ability to resist microcutting was enhanced; (2) after extrusion from the matrix, Grp spread to the wear surface to form a graphite-rich surface film, preventing severe adhesion and ploughing. As the volume fraction of Grp increased to 10 vol %, the wear rate of the composite increased. This was because the continued addition of Grp did not further increase the hardness of the composite, and the interface between Grp and the matrix was usually the source of the crack. These greatly reduced the plasticity of the composite itself, and decreased the ability of the composite surface to resist spalling and wear. Under the action of cyclic load, the shear deformation of the surface layer accumulated continuously, and the shear band at a certain depth below the surface layer expanded into a crack. The expansion of the crack in the depth direction was hindered by the normal stress parallel to the surface, and at this depth, the crack expanded in a direction that was parallel to the surface, until it reached the wear surface. Figure 8b is the curve of the friction coefficient of the as-cast AZ91 alloy and Grp/AZ91 composites over time. It shows that the friction coefficient of the as-cast AZ91 alloy obviously increased with the increase of friction time. This was because, in the absence of effective lubrication, the rough peak on the surface of the softer composite gradually disappeared with the increase of friction time, and the friction surface became flatter, so that the real contact area between the AZ91 alloy and the mating surface increased, and the friction coefficient increased. After the addition of 5 vol % of Grp, the friction coefficient of the Grp/AZ91 composites decreases significantly, indicating that the introduction of graphite particles could actually reduce the friction coefficient and improve the lubrication condition of the friction pair. 

The average friction coefficient of the Grp/AZ91 composites slight decreased when the volume fraction of Grp increased to 10 vol %. This was due to the following reasons: (1) The lubrication conditions of friction pairs were improved and the friction coefficient was reduced with the increase of Grp. (2) As mentioned above, the ability of material surface to resist exfoliation and wear decreased, and the real contact area of material and spouse surface increased with the increase of Grp, resulting in the increase of the friction coefficient. As a result of these two aspects, the average friction coefficient of the composites decreases slightly when the Grp content increases from 5% to 10%.

Rohatgi et al. studied graphite reinforced composites with different matrices [33]. The results showed that the friction coefficient of the composites decreased with the increase of graphite content when the content of graphite particles was less than 20 vol %. The friction coefficient of the composites was stable at 0.2, which was about the friction coefficient of graphite when the content of graphite particles was more than 20 vol %. In this experiment, the addition of Grp was not up to 20%, as mentioned in Rohatgi’s research, so the friction coefficient decreases with the addition of Grp. However, the friction coefficient decreased obviously when the content of Grp reached 5%. The decreasing degree of the friction coefficient became weaker when the content of Grp reached 10%.

**Figure 9:**
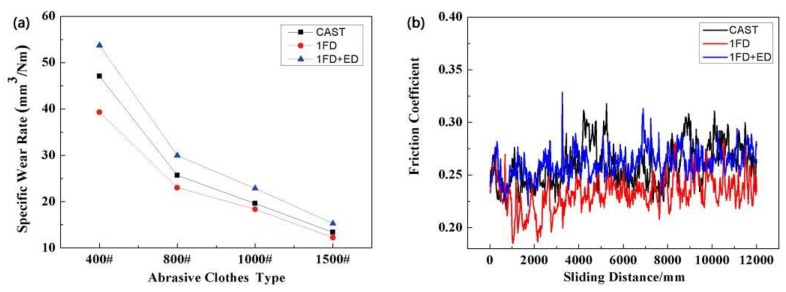
(**a**) Wear rate under different cloth abrasiveness types, and (**b**) friction coefficient versus time of the 5% Grp/AZ91 composites after hot deformation.

Through the above analysis, it was found that the 5% Grp/AZ91 composites had the best performance. In order to further study the effects of different thermal deformation modes on the wear resistance of composites, the 5% Grp/AZ91 composites after forging once, and 5% Grp/AZ91 composites after forging plus extrusion once were selected, and their wear rate and friction coefficients were tested. The results were compared with those of the as-cast Grp/AZ91 composites. Figure 9a shows the wear rate of the 5% Grp/AZ91 composites under different abrasive cloth models. It shows that as the abrasive cloth grit decreased, the wear rate of the as-cast Grp/AZ91 composites, the 5% Grp/AZ91 composites after forging once, and the 5% Grp/AZ91 composites after forging plus extrusion once all decreased. This was because the smaller the size of the abrasive cloth grit, the less likely it was to scratch the surface of the specimen.

By a longitudinal comparison of the three composites at different states, it was found that the wear rates of the 5% Grp/AZ91 composites after forging once decreased slightly, while the wear rates of the composites increased after hot extrusion. It could be seen that the change rule of the wear resistances of the 5% Grp/AZ91 composites after thermal deformation was contrary to the change trend of the mechanical properties of the composite. In this test, the wear resistances were the worst in the as-extruded composites with the highest hardnesses, and the reasons for these phenomena were: (1) The decrease in the size of Grp. After hot extrusion, the size of Grp decreased obviously, while a smaller Grp tended to peel off with the matrix during ploughing, and it was wrapped in wear debris, making it difficult to play a role in lubrication. After hot extrusion, the bonding strength of Grp to the matrix increased, and this also limited the lubrication of Grp. (2) The increase in the temperature of the wear surface. In the test, there were small loads and small sliding speeds, but there might still be a high temperature on the friction surface due to the wear of the abrasive cloths, thus weakening the hardness improvement caused by the work hardening and the fine-grain strengthening of the surface of the composite. (3) The distribution orientation of Grp in the 5% Grp/AZ91 composites after forging plus one round of extrusion was perpendicular to the friction pair. The vertical orientation of Grp in the composite after hot extrusion led to the fact that the actual volume fraction of the particles near the surface of the composite was lower than the overall volume fraction of the composite. Therefore, in conclusion, compared with the as-cast composite, the improvement of the wear resistance of the composite after forging once could not be attributed to significant decrease in the size of Grp after forging, and a significant improvement in the average microhardness and plasticity. The decrease in the wear resistance of the composite after forging plus extrusion once was due to a combination of a significant decrease in the size of Grp, high temperature on the wear surface, and the vertical orientation distribution of Grp after extrusion.

Figure 9b is the curve of the friction coefficient of the as-cast Grp/AZ91 composites and 5% Grp/AZ91 composites after thermal deformation. It shows that the friction coefficient of the 5% Grp/AZ91 composites after forging once was decreased, compared with the friction coefficient of the as-cast composites, and this was due to slight adhesive wear on the surface of the as-cast composite. After hot extrusion, the friction coefficient of the 5% Grp/AZ91 composites increased significantly. This can be attributed to the small Grp in the as-extruded 5% Grp/AZ91. The small Grp did not play a role in lubrication after matrix peeling, and the content of Grp near the surface was lower than the actual content because of the vertical orientation.

**Figure 10:**
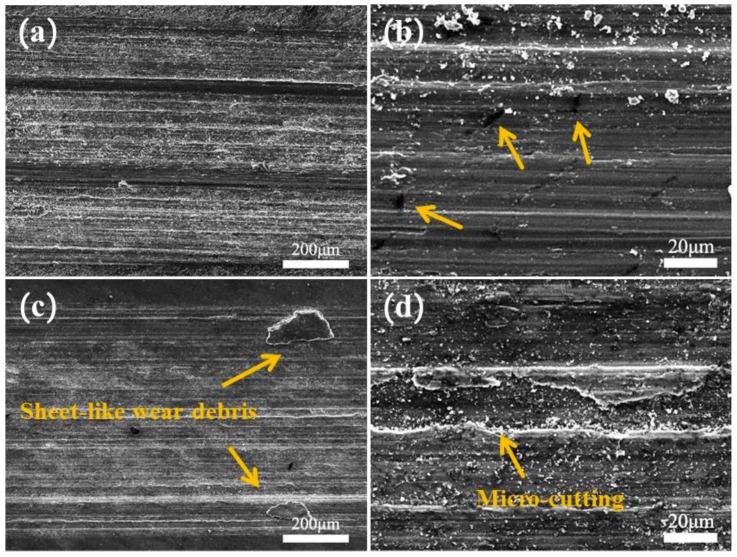
Low- (**a**,**c**) and high- (**b**,**d**) magnification SEM images of the worn surface of the 5% Grp/AZ91 composites after hot deformation: 1FD (**a**,**b**); 1FD+ED (**c**,**d**).

Figure 10 shows the SEM morphology of the wear surface of 5% Grp/AZ91 after thermal deformation. From Figure 10a, it can be found that the composites after forging once were relatively flat over the whole wear surface, and there was an obvious ploughing morphology. The exposure of Grp could be observed from high-magnification SEM (Figure 10b) of the composite, and this indicated that the wear surface was well-lubricated. Therefore, the wear mechanism of the 5% Grp/AZ91 composites after forging once was mainly due to ploughing wear. Figure 10c shows that large flaky wear debris was evident on the wear surface of 5% Grp/AZ91 after forging plus extrusion once, which could be called wedge formation, and deep micro-cutting grooves were found in the high-magnification SEM (Figure 10d). Compared with the situation where composites on both sides of ploughing were pushed by the ploughing direct cutting of the composite by micro-cutting, this makes the wear of micro-cutting greater and the friction coefficient higher. The main wear mechanisms of the as-extruded 5% Grp/AZ91 composites were wedge formation and micro-cutting wear.

## 4. Conclusions

(1) After 5 vol % Grp is added into the as-cast AZ91, the Mg17Al12 phase is no longer precipitated reticularly along the grain boundary, the Al4C3 phase is newly formed in the composite, and the grain size decreases, compared with the grain size of the composite. As the volume fraction of Grp increases, the grains of the composite are gradually refined.
(2) Compared with the grain size and the Grp size of the as-cast composite, the grain size and the Grp size of the 5% Grp/AZ91 after forging once are significantly reduced. With the increase of forging passes, the grain size first decreases and then increases, and the Grp size gradually decreases. With the increase of forging passes, there is little change in yield strength, while the tensile strength and elongation increase gradually.
(3) Compared with the grain size and Grp size of the composite after forging, the grain size and the Grp size of the 5% Grp/AZ91 after forging plus extrusion once are further reduced. With the increase of forging passes before extrusion, the grain size decreases gradually and the Grp size remains unchanged. With the increase of forging passes before extrusion, the yield strength, tensile strength, and elongation increase slightly.
(4) For the 5% Grp/AZ91 after forging once, the wear rate decreases, the friction coefficient decreases, and the wear mechanism is mainly slight abrasive wear, compared with those of the as-cast composite. For the 5% Grp/AZ91 after forging plus extrusion once, the wear rate increases, the friction coefficient increases, and the wear morphology tends to be serious compared with those of the composite after forging once. The wear mechanisms of the as-extruded composite are wedge formation and micro-cutting wear.

## Figures and Tables

**Table 1:** Composition of AZ91 alloy (wt %).

Mg	Al	Zn	Mn	Si
89.68	9.35	0.7	0.25	0.02

**Table 2:** Chemical composition of the covering agent (wt %).

Brand	Main Components (wt %)					Impurities (≤wt %)		
	KCl	NaCl	CaCl	BaCl	NaCl + CaCl	Insoluble Substance	MgO	H_2_O
RJ-6	46–54	1.5–2.5	2.7–2.9	28–31	8	1.5	1.5	2

**Table 3:** The Grp sizes of 5% Grp/AZ91 composites after MDF (μm).

	1FD	3FD	6FD
Grp size	4.8 ± 9.6	3.2 ± 11.1	2.2 ± 7.2

**Table 4:** The grain sizes of 5% Grp/AZ91 composites after MDF (μm).

	1FD	3FD	6FD
Grain size	5.9 ± 10.2	4.0 ± 10.5	5.2 ± 9.0

**Table 5:** The Grp sizes of 5% Grp/AZ91 composites after hot deformation (μm).

	1FD+ED	3FD+ED	6FD+ED
Grp size	1.8 ± 4.7	1.7 ± 5.1	1.8 ± 6.4

**Table 6:** The grain sizes of 5% Grp/AZ91 composites after multistep deformation (μm).

	1FD+ED	3FD+ED	6FD+ED
Grain size	1.5 ± 4.4	1.5 ± 7.3	1.3 ± 5.2

**Table 7:** The mechanical properties of the 5% Grp/AZ91 composites after MDF.

	YS/MPa	UTS/MPa	Elongation/%	Hardness/Hv
1FD	203 ± 27	257 ± 6	1.7 ± 0.4	117.9 ± 8.1
3FD	204 ± 19	273 ± 9	2.0 ± 0.4	131.8 ± 26.6
6FD	207 ± 14	278 ± 6	2.6 ± 0.6	125.4 ± 6.9

**Table 8:** The mechanical properties of the 5% Grp/AZ91 composites after multistep deformation.

	YS/MPa	UTS/MPa	Elongation/%	Hardness/Hv
1FD+ED	269 ± 21	360 ± 11	3.5 ± 0.5	147.7 ± 22.1
3FD+ED	272 ± 4	361 ± 6	4.0 ± 0.3	152.8 ± 29.3
6FD+ED	276 ± 22	371 ± 10	4.3 ± 1.0	154.4 ± 12.4
